# Retracted: The Copper Radioisotopes: A Systematic Review with Special Interest to ^64^Cu

**DOI:** 10.1155/2018/3860745

**Published:** 2018-07-08

**Authors:** BioMed Research International

BioMed Research International has retracted the article titled “The Copper Radioisotopes: A Systematic Review with Special Interest to ^64^Cu” [1]. The article was found to contain a substantial amount of material from the following published articles:

- Szymański et al. [2], which is cited as reference [4].
- Wadas et al. [3], which is cited as reference [3].
- Carolyn and Ferdani [4], which is cited as reference [54].
- Smith et al. [5], which is cited as reference [34].
